# Use of electronic immunization registry in the surveillance of adverse events following immunization

**DOI:** 10.11606/S1518-8787.2018052000295

**Published:** 2018-01-29

**Authors:** Ana Paula Sayuri Sato, Vinícius Leati de Rossi Ferreira, Márcia de Cantuária Tauil, Laura Cunha Rodrigues, Mariana Bernardes Barros, Edmar Martineli, Ângela Aparecida Costa, Marta Inenami, Eliseu Alves Waldman

**Affiliations:** I Universidade de São Paulo. Faculdade de Saúde Pública. Departamento de Epidemiologia. São Paulo, SP, Brasil; II Faculty of Epidemiology and Population Health. London School of Hygiene and Tropical Medicine. London, UK; III Universidade de São Paulo. Centro de Tecnologia da Informação de São Carlos. São Carlos, SP, Brasil; IV Universidade de São Paulo. Faculdade de Saúde Pública. Serviço Especial de Saúde de Araraquara. Araraquara, SP, Brasil

**Keywords:** Vaccines, adverse effects, Electronic Health Records, utilization, Immunization Programs, Epidemiological Surveillance, Vacinas, efeitos adversos, Registros Eletrônicos de Saúde, utilização, Programas de Imunização, Vigilância Epidemiológica

## Abstract

**OBJECTIVE:**

To describe adverse events following vaccination (AEFV) of children under two years old and analyze trend of this events from 2000 to 2013, in the city of Araraquara (SP), Brazil.

**METHODS:**

This is a descriptive study conducted with data of the passive surveillance system of AEFV that is available in the electronic immunization registry (EIR) of the computerized medical record of the municipal health service (Juarez System). The study variables were: age, gender, vaccine, dose, clinical manifestations and hospitalization. We estimated rates using AEFV as numerator and administered doses of vaccines as denominator. The surveillance sensitivity was estimated by applying the method proposed by the Centers for Disease Control and Prevention. We used Prais-Winsten regression with a significance level of 5.0%.

**RESULTS:**

The average annual rate of AEFV was 11.3/10,000 administered doses, however without a trend in the study period (p=0.491). Most cases occurred after the first dose (41.7%) and among children under one year of age (72.6%). Vaccines with *pertussis* component, yellow fever and measles-mumps-rubella were the most reactogenic. We highlighted the rates of hypotonic-hyporesponsive episodes and convulsion that were 4.1/10,000 and 1.5/10,000 doses of vaccines with *pertussis* component, respectively, most frequently in the first dose; 60,0% of cases presented symptoms in the first 24 hours after vaccination, however, 18.6% showed after 96 hours. The sensitivity of surveillance was 71.9% and 78.9% for hypotonic-hyporesponsive episodes and convulsion, respectively.

**CONCLUSIONS:**

The EIR-based AEFV surveillance system proved to be useful and highly sensitive to describe the safety profile of vaccines in a medium-sized city. It was also shown that the significant increase of the vaccines included in the basic vaccination schedule in childhood in the last decade did not alter the high safety standard of the National Immunization Program.

## RESUMO

**OBJETIVO:**

Descrever os eventos adversos pós-vacina ocorridos em crianças com até dois anos de idade e analisar a tendência desses eventos, entre 2000 e 2013, em Araraquara, SP, Brasil.

**MÉTODOS:**

Estudo descritivo com dados da vigilância passiva de eventos adversos pós-vacina, disponíveis no registro informatizado de imunização do prontuário eletrônico do serviço municipal de saúde (Sistema Juarez). As variáveis de estudo foram: idade, sexo, vacina, dose, manifestações clínicas e hospitalização. As taxas foram estimadas tomando-se os casos de eventos adversos pós-vacina como numerador e as doses aplicadas, como denominador. Estimou-se a sensibilidade da vigilância de eventos adversos pós-vacina aplicando-se o método proposto pelo *Centers for Disease Control and Prevention*. Para análise de tendência utilizou-se a regressão de Prais-Winsten, considerando o nível de significância de 5,0%.

**RESULTADOS:**

A taxa anual média de eventos adversos pós-vacina foi de 11,3/10.000 doses aplicadas, porém, sem tendência no período estudado (p = 0,491). Houve predomínio de casos após a primeira dose (41,7%) e entre menores de um ano de idade (72,6%). As vacinas com componente *pertussis*, de febre amarela e de sarampo-caxumba-rubéola foram as mais reatogênicas. Destacou-se a frequência de episódio hipotônico hiporresponsivo e convulsão, com taxas de 4,1/10.000 e 1,5/10.000 doses de vacinas com componente *pertussis*, respectivamente, mais elevadas na primeira dose; 60,0% dos casos apresentaram sintomas nas primeiras 24 horas após a vacinação e 18,6% apresentaram-se após 96 horas. A sensibilidade da vigilância foi estimada em 71,9% e 78,9% para episódio hipotônico hiporresponsivo e convulsão, respectivamente.

**CONCLUSÕES:**

O sistema de vigilância de eventos adversos pós-vacina com base no registro informatizado de imunização mostrou-se útil e dotado de elevada sensibilidade para descrever o perfil de segurança das vacinas em um município de médio porte. Adicionalmente, a expressiva ampliação, na última década, das vacinas incluídas no esquema básico de vacinação na infância não alterou o elevado padrão de segurança do Programa Nacional de Imunizações.

## INTRODUCTION

Vaccination actions with the National Immunization Program (NIP) have contributed to a significant reduction in the burden of vaccine-preventable diseases in Brazil^8^. As the risk perception of these diseases decreases as a result of successful immunization programs, the perceived risk of vaccine-preventable diseases (adverse events following immunization – AEFI) may increase, decreasing adherence to vaccination and, consequently, creating conditions for resurgence of diseases already controlled^4^.

In 2003, the World Health Organization (WHO) established a comprehensive surveillance system to ensure the safety of vaccines administered in national immunization programs^22^. In Brazil, the state of São Paulo evaluates the AEFI since 1984, an initiative that became national in 1998 with the implementation of the passive surveillance system of AEFI by the NIP. The surveillance of AEFI is aimed at correcting programmatic errors, identifying the existence of specific more reactive batches, investigating rare or unknown events, and studying associated factors to maintain the public confidence in the immunization program^22^.

Currently, the Information System of the NIP (IS-NIP) is in the process of being implemented in Brazil, which is a database on vaccination that also aggregates the Information System of AEFI (IS-AEFI), with online record on the notification, investigation, management, and final classification of these events.

Since 1987, the municipality of Araraquara (state of São Paulo) has an electronic immunization registry (EIR), in which individual vaccination data and notifications on AEFI are stored, from the initiative of the Special Health Service of Araraquara (SESA) of the Faculdade de Saúde Pública of the Universidade de São Paulo and technical support of the Information Technology Center of São Carlos (CeTI-SC). This EIR is one of the components of the electronic medical record called Juarez System and the first EIR in Brazil; however, despite almost three decades of use, it has been the subject of few publications. Its evaluation is useful to provide subsidies to other municipal EIR that develop similar initiatives, as well as to the IS-AEFI of the NIP.

Thus, this study aimed to describe the AEFI in children aged ≤ 24 months and analyze their tendency, in the municipality of Araraquara, from 2000 to 2013, using the EIR of the Juarez System.

## METHODS

This is a descriptive study with a trend analysis focused on the frequency and distribution of cases reported and confirmed in the passive surveillance system of AEFI in children up to 24 months of age living in the municipality of Araraquara, state of São Paulo, Brazil, from 2000 to 2013.

The municipality of Araraquara has a population of approximately 210,000 inhabitants, with approximately 3,000 live births per year; 97.2% of its population is urban and the Municipal Human Development Index is 0.815. In relation to health equipment, Araraquara has 11 traditional primary health units, 16 units with Family Health Strategy (coverage of approximately 40.0% of the population), three general hospitals, two emergency units, one psychiatric hospital, and one specialty outpatient clinic. The municipality has a successful vaccination program and has high vaccine coverage, so that vaccine-preventable diseases have been controlled since the 1990s^a^.

The SESA is responsible for the management of the electronic medical record of the municipal health system (Juarez System), which includes the EIR as one of its components, which can identify individuals with late vaccines and, therefore, summon them to update it. The EIR of the Juarez System is used by all health services in Araraquara, with online access since 2012, so that health professionals can verify and record vaccination actions, including AEFI notifications, in real time. Before 2012, the record of AEFI was carried out exclusively by the staff (nurses, nursing technicians, and administrative personnel) of the SESA. Currently, the SESA still receives, verifies, and stores all the AEFI notification records of the municipality of Araraquara and registers the notification in the Juarez System whenever this procedure is not carried out by the service unit.

The data from this research were collected from the passive surveillance system of AEFI of the municipality of Araraquara, and they are available in the database of the Juarez System (date of birth, sex, date of vaccination, date of onset of symptoms, vaccine, and dose). Data on clinical manifestations and hospitalization were only reported in the notification records of AEFI, which were searched in order to supplement information that was missing in the Juarez System.

For the confirmation of cases, the municipal surveillance of AEFI observed the specific criteria for each type of event established in a technical document of the NIP – Ministry of Health^b^. Severe AEFI were evaluated and confirmed by one physician of the SESA, and the other types were evaluated and confirmed by professionals of the network of health services of the municipality.

In this study, after analyzing the notification records, the confirmed cases of AEFI were classified as events with local or systemic manifestations. On the notification of more than one AEFI for the same child and dose of vaccine, we considered them as a single case with two or fewer events.

The vaccines used during the study period generally had the same origin, most of which were produced in Brazil by the *Instituto de Tecnologia em Imunobiológicos Bio-Manguinhos* – FIOCRUZ (diphtheria-tetanus-pertussis (whole cell) [DTPw], *Haemophilus influenzae b*, tetravalent, yellow fever, 10-valent pneumococcal conjugate, oral and inactivated polio, rotavirus, and measles-mumps-rubella [MMR]), *Instituto Butantan* (DTPw, hepatitis B, and influenza), and *Fundação Ataulpho de Paiva* (BCG). The yellow fever vaccine is based on a live, attenuated viral strain, 17D or equivalent, grown on embryonated chicken eggs. The most recently introduced vaccines in the Brazilian calendar follow the processes of technology transfer.

The method proposed by the Centers for Disease Control and Prevention was used to estimate the sensitivity of the surveillance of AEFI in Araraquara^3^. For this purpose, we used the rates of hypotonic-hyporesponsive episodes and seizure after pertussis vaccine calculated in this study comparing them with those obtained by the study of Martins et al.^18^, which was considered as a reference for this comparison (gold standard). The aforementioned study^18^ has estimated the incidence of these events from the active surveillance of 21,064 children within 48 hours after administering the diphtheria-tetanus-pertussis (whole cell) and *Haemophilus influenzae b* vaccines at health centers in the city of Rio de Janeiro, Brazil. Martins et al.^18^ have used a vaccine of the same origin as that administered in Araraquara during the period of this study.

The data were entered into the database and then analyzed using the software Stata 13. We estimated the rates of AEFI cases using the cases of adverse events as numerator and the number of doses of vaccine administered as denominator. We also estimated the ratio between the number of doses administered and the occurrence of a case by dividing the number of doses by the number of AEFI cases.

The trend analysis of the rates of AEFI was performed using a linear regression model for time series with the Prais-Winsten method, in order to minimize the first order autocorrelation of residuals^1^. The dependent variable was the rate of AEFI cases, and the independent variable was the calendar year. We considered a significance level of 5%, and we carried out a residue analysis to verify the suitability of the final model.

This study was approved by the Research Ethics Committee of the Faculdade de Saúde Pública of the Universidade de São Paulo (Process 29516814.1.0000.5421), according to the recommendations of Resolution 466 of 2012 – National Health Council for Scientific Research in Human Beings.

## RESULTS

From 2000 to 2013, 42,735 children living in Araraquara were registered in the Juarez System, with an average of 3,025 births per year. In this period, 763,548 doses of vaccines were administered. The number of doses administered per year in children up to 24 months of age ranged from 40,611 in 2007 to 77,247 in 2011.

**Figure 1:**
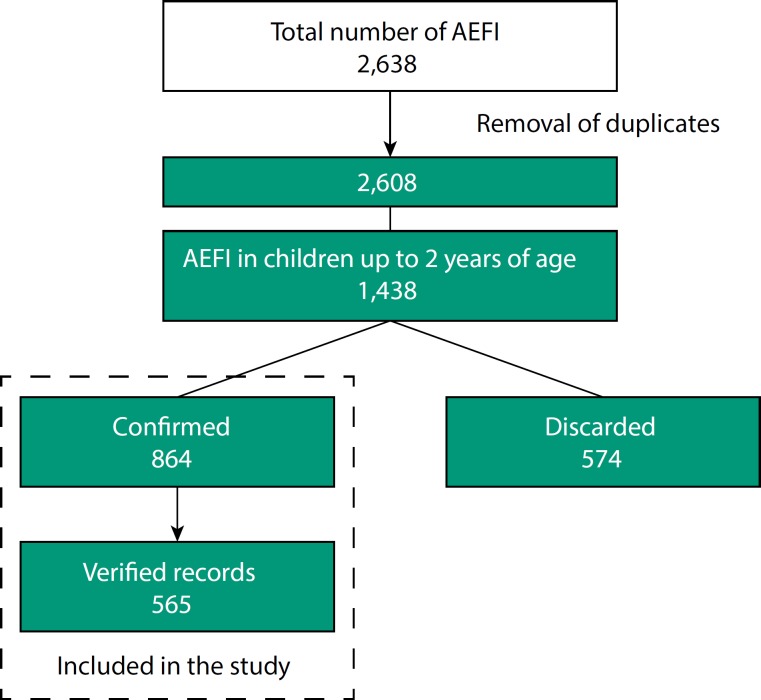
Scheme of the selection of AEFI cases recorded in the Juarez System and included in the study. Araraquara, state of São Paulo, Brazil, 2000 to 2013. AEFI: adverse events following immunization

Figure 1 presents the scheme of the selection of AEFI cases recorded in the Juarez System and included in this study. We identified 2,638 notifications in the period from 2000 to 2013 and, after the analysis of the consistency of the database (removal of duplicates), we considered 2,608 notifications among persons of all age groups. We found 1,438 reports for children up to 24 months of age, of which 864 (60.1%) were confirmed cases (mean annual rate of 11.3/10,000 doses of any vaccine, with no trend – p = 0.491), with 54.0% of the cases being in males and 72.6% in children under 12 months of age. Of the confirmed cases, 360 (41.7%) occurred after the first dose of the vaccine.

**Figure 2:**
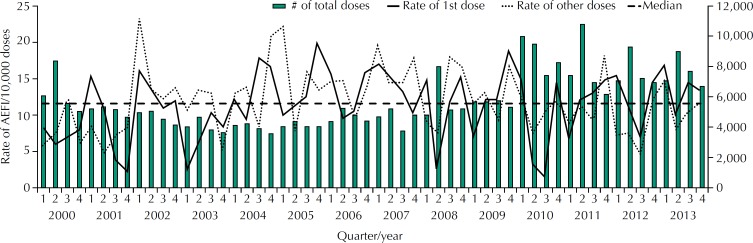
Distribution of the number of doses administered and rates of AEFI cases in the first dose and other doses per 10,000 doses, according to quarter/year of occurrence in children up to 24 months of age. Araraquara, state of São Paulo, Brazil, 2000 to 2013. (n = 864) AEFI: adverse events following immunization

Figure 2 shows the distribution by quarter/year of the number of doses administered and the rates of AEFI cases per 10,000 doses, specifying whether the dose administered was the first one or the other ones. In general, the second quarter of each year has the highest number of doses administered. There was an increasing trend in the number of doses administered (p = 0.041), especially after 2010, when two new routine vaccines (meningococcal C conjugate vaccine and 10-valent pneumococcal conjugate vaccine) and one campaign vaccine for influenza were introduced. The rates of AEFI cases for both the first dose and the other doses showed seasonal variation.

For 565/864 (65.4%) AEFI cases, we could find the notification records, which were manually analyzed, focusing on information regarding the clinical manifestations and hospitalizations of the cases. There was no difference in sex and age distribution between those with and without a verified record. The 565 cases with analyzed records had 1,251 clinical manifestations (median = two manifestations per case), of which 419 (33.5%) were local and 832 (66.5%) were systemic.

Among the vaccines most frequently associated with AEFI cases, we highlight those with pertussis (whole-cell) (DTPw, DTPw-Hib [tetravalent], and DTPw-Hib-Hepatitis B [pentavalent]), with a rate of approximately 25/10,000 doses, the yellow fever vaccine, with a rate of approximately 6.9/10,000 doses, the MMR vaccine, with a rate of approximately 11.1/10,000 doses, and the MMRV vaccine, with a rate of approximately 14.1/10,000 doses (Table 1).

**Table 1:** Distribution of AEFI cases, number of doses administered, number of doses per case, and rate per 10,000 doses, according to vaccine and route of administration in children up to 24 months of age. Araraquara, state of São Paulo, Brazil, 2000 to 2013. (n = 565)

Variable		Number of AEFI cases	Total of doses	Number of doses per case	Rate per 10,000 doses
Vaccine					
	BCG	41	42,767	1,043	9.6
	Hepatitis B	25	120,819	4,833	2.1
	DTPw-Hib and Pentavalent^a^	252	102,689	407	24.5
	DTPw	161	62,980	391	25.6
	10/13V Pneumococcal Conjugate	28	45,903	1,639	6.1
	Men-C-ACYW	17	36,289	2,135	4.7
	IPV^b^	7	8,634	1,233	8.1
	OPV^c^	47	158,367	3,369	3.0
	Rotavirus	38	42,974	1,131	8.8
	MMR	53	47,718	900	11.1
	MMRV^d^	1	711	711	14.1
	Influenza	15	26,152	1,743	5.7
	Yellow fever	14	20,170	1,441	6.9
Route of administration^e^					
	Intramuscular	505	403,466	799	12.5
	Intradermal	41	42,767	1,043	9.6
	Subcutaneous	68	68,599	1,009	9.9
	Oral	85	201,341	2,369	4.2

a DTPw-Hib or tetravalent: diphtheria-tetanus-pertussis (whole-cell) and *Haemophilus influenzae b*. Pentavalent: diphtheria-tetanus-pertussis (whole-cell) and *Haemophilus influenzae b* and Hepatitis B.

b IPV: inactivated polio vaccine.

c OPV: oral polio vaccine.

d MMRV: measles, mumps, rubella, and varicella.

e The same individual may have more than one vaccine associated with AEFI.

Among the 14 AEFI cases of yellow fever, 13 (92.8%) happened in children up to 12 months of age. The main clinical manifestations were fever, exanthema, and hypersensitivity, and there was no hospitalization. Regarding the MMR vaccine, 53 AEFI cases were reported, of which 50 (94.3%) occurred in children aged 12–24 months of age and 51 (96.2%) in children with primary vaccination. Of these 53 cases, 25 (47.2%) had exanthema, 20 (37.7%) parotitis, 27 (50.9%) fever, and six (11.3%) adenopathy (data not presented in tables), and we highlight that a single case may have presented more than one event.

Among the clinical manifestations, local reactions were frequent in all vaccines, with an overall rate of 5.5/10,000 doses, including all vaccines (1:1,825 doses), ranging from 12.0/10,000 doses of pertussis vaccines to 1.0/10,000 doses of yellow fever vaccine. Pain (2.5/10,000 doses) and nodule (2.1/10,000 doses) were the most frequent local reactions (Table 2).

**Table 2:** Clinical manifestations (n = 1,251^a^) and hospitalization of adverse effects following immunization, number of doses per event, and rate per 10,000 doses, in children up to 24 months of age. Araraquara, state of São Paulo, Brazil, 2000 to 2013.

Clinical manifestations/Hospitalization		Number of events	Number of doses per event	Rate per 10,000 doses^b^
Local				
	Pain	189	4,040	2.5
	Nodule	163	4,684	2.1
	Abscess	33	23,138	0.4
	Lymphadenitis	33	23,138	0.4
	Ulcer	1	763,548	0.1
Systemic				
	Headache	1	763,548	0.1
	Nausea	10	76,355	0.1
	Diarrhea	23	33,198	0.3
	Vomiting	54	14,140	0.7
	Cyanosis	27	28,280	0.4
	Malaise	23	33,198	0.3
	Irritability	102	7,486	1.3
	Sleepiness	44	17,353	0.6
	Myalgia	7	109,078	0.1
	Exanthema	46	16,599	0.6
	Jaundice	1	763,548	0.1
	Parotitis	20	38,177	0.3
	Fever	344	2,220	4.5
	< 38°C	51	14,971	0.7
	38|-|39,4°C	196	3,896	2,6
	≥ 39.5°C	68	11,229	0.9
	Hypotonic-hyporesponsive episode	67	11,396	0.9
	Adenoiditis	18	42,419	0.2
	Arthralgia	1	763,548	0.1
	Hypersensitivity	11	69,413	0.1
	Seizure	25	30,542	0.3
	Loss of consciousness	6	127,258	0.1
	Myelitis	1	763,548	0.1
	Paresis	1	763,548	0.1
Hospitalization		16	47,722	0.2

a The 565 cases for which we had information presented 1,251 types of clinical manifestation.

b Rate of AEFI for dose of any vaccine.

Among the systemic reactions, the following stand out: fever (rate of 4.5/10,000 doses, including all vaccines), hypotonic-hyporesponsive episode (HHE) (rate of 4.1/10,000 doses of pertussis vaccines), and parotitis (rate of 4.2/10,000 doses of measles-mumps-rubella vaccine). The seizure rate was 1.5/10,000 doses of pertussis vaccine and the hospitalization rate was 0.2/10,000 doses, including all vaccines (sixteen children), mainly associated with HHE and seizure (Table 2).

Regarding the occurrence of HHE (n = 67), there was no difference according to sex. Children younger than 12 months of age had a higher frequency (n = 54, 80.6%), especially after the first dose of the pertussis vaccine (n = 29, 43.3%). Seizures (n = 25) were more frequent in male children and after pertussis vaccine and first booster with DTPw, and eight (32.0%) cases were hospitalized. The median age of occurrence for both events was four months (Table 3).

**Table 3:** Distribution of hypotonic-hyporesponsive events and seizures in children up to 24 months of age who received pertussis vaccine, according to sex, age, dose administered, and hospitalization. Araraquara, state of São Paulo, Brazil, 2000 to 2013.

Variable		HHE			Seizures		
		n (%)	Rate per 10,000 doses*	Doses per event	n (%)	Rate per 10,000 doses*	Doses per event
Sex							
	Male	33 (49.3)	3.9	2,530	17 (68.0)	2.0	4,911
	Female	34 (50.7)	4.1	2,418	8 (32.0)	1.0	10,274
Age							
	< 12 months	54 (80.6)	4.3	2,320	25 (100)	2.0	5,011
	12|-|24 months	13 (19.4)	3.2	3,106	0 (0)	-	-
	Median	4 months			4 months		
Dose administered							
	1st dose	29 (43.3)	6.9	1,451	2 (8.0)	0.5	21,041
	2nd dose	16 (23.9)	3.8	2,634	7 (28.0)	1.7	6,020
	3rd dose	13 (19.4)	3.1	3,218	7 (28.0)	1.7	5,977
	1st booster	9 (13.4)	2.3	4,400	9 (36.0)	2.3	4,400
Hospitalization		7 (10.5)	0.4	23,667	8 (32.0)	0.5	20,709
Total		67	4.1	2,473	25	1.5	6,626

HHE: hypotonic-hyporesponsive episode

* Rate of AEFI per dose of pertussis vaccine (total number of doses = 165,669; number of males = 83,477; number of females = 82,192; number of < 12 months = 125,291; number of 12|-| 24 months = 40,378; number of 1st doses = 42,082; number of 2nd doses = 42,143; number of 3rd doses = 41,839; number of 1st boosters = 39,605).

In order to estimate the sensitivity of the surveillance of AEFI of Araraquara, we compared the rates of HHE and seizure (5.7/10,000 and 1.9/10,000 doses of tetravalent vaccine, respectively) in a research published by Martins et al.^18^ with the rates identified in this study (4.1/10,000 and 1.5/10,000 doses of pertussis vaccine, respectively). Thus, the sensitivity found was 71.9% for HHE and 78.9% for seizures.

Regarding the time to onset of the first symptoms, we found 540 cases with this information, of which 60.0% (n = 324) presented signs and symptoms in the first 24 hours after vaccination. This proportion increased to 82.0% in cases of HHE and seizure. The cases of late AEFI, i.e. those that occurred 96 hours after vaccination, accounted for 18.6% (n = 100) of the total studied, with emphasis on events following the MMR (n = 31), BCG (n = 28), and tetravalent vaccines (DTPw-Hib) (n = 11) (data not shown in tables).

## DISCUSSION

The EIR has been used for decades by national immunization programs in developed countries that have become very complex because of the diversity of vaccines and schedules used routinely^4^ ^,^ ^5^. In these countries, the EIR is an important instrument for the promotion of high and homogeneous vaccination coverage, as well as for the monitoring of immunobiological safety^2^ ^,^ ^22^.

This study, to the best of our knowledge, is the first one done in Brazil, analyzing data from an EIR for the description of AEFI. It is especially timely, as it provides results that can provide support for the current proposal by the NIP of implementing an EIR articulated with an information system for AEFI.

Several of its results are relevant, among them, the significant increase of doses administered from the rapid expansion of the types of vaccines included in the basic vaccination schedule, including in the combined form^8^. On the other hand, the stability of the rates of AEFI cases, verified in the period, suggests that the greater complexity achieved by the NIP, resulting from changes in the basic immunization schedule, did not change the safety profile of the vaccines included in the NIP schedule.

An interesting result is the clear seasonal variation of the rates of AEFI cases found in this study. It is worth noting that it is not the result of the use of batches of more reatogenic vaccines, since we did not identify changes in the mean annual rates of AEFI. In fact, it reflects the sharp rise in the number of doses administered in annual vaccination campaigns (such as polio and influenza, the latter after 2010) or specific campaigns (such as the measles follow-up campaign in 2000), consequently determining a greater concentration of the number of AEFI in time. Similar behavior has been found in several studies, including some in Brazil^9^ ^,^ ^11^ ^,^ ^12^. This increase in AEFI in a short period of time usually creates a warning to health professionals, increasing the sensitivity of the surveillance^22^.

This study also showed the prevalence of AEFI in the first year of life and higher rates of AEFI associated with the pertussis (whole cell), MMR, and yellow fever vaccines. These results are consistent with the literature, which indicates a higher frequency of HHE and seizure after the administration of the pertussis vaccine, parotitis after the measles-mumps-rubella vaccine, and reactions such as fever and exanthema after the yellow fever vaccine ^10^ ^,^ ^13^ ^,^ ^15^ ^,^ ^18^. In turn, considering that the risk of AEFI is higher in the first dose^6^ ^,^ ^13^ ^,^ ^18^ and that most vaccines are concentrated in the first months of life, the predominance of AEFI in this age group is explained.

An important fact was the high sensitivity of the surveillance of AEFI of Araraquara, at 71.9% for HHE and 78.9% for seizure. A Brazilian study that carried out this evaluation, considering the same study as the “gold standard”^18^, has found a mean sensitivity of 22.3% and 31.6% for HHE and seizure, respectively, with a high variation among states (approximately 3.0% to 100.0% for HHE and 5.0% to 90.0% for seizures)^19^. In this way, we can see the potential of the EIR of the Juarez System in the surveillance of AEFI.

The disadvantages of this type of passive surveillance of AEFI are partially compensated by the use of the EIR as a source. In addition to having a more precise numerator and denominator for the estimates of incidence of AEFI, the EIR has information on the child's previous history of vaccination and simultaneous exposure to multiple vaccines^22^. In addition, the EIR allows a more accurate description of AEFI as it records the date of onset of the symptoms, allowing the identification of late AEFI. In turn, the indication or not of revaccination of individuals who had AEFI in the first dose can be evaluated.

An additional advantage of the EIR is the possibility to implement active surveillance systems when the EIR is linked to an electronic record^5^ ^,^ ^14^, similarly to the Vaccine Safety Datalink of the United States^23^, or define and detect sentinel AEFI^7^. Moreover, the EIR can be used to generate hypotheses in epidemiological studies and in the articulation with other databases^2^ ^,^ ^5^ ^,^ ^16^ ^,^ ^17^ ^,^ ^20^ ^,^ ^21^.

This study has limitations regarding the use of secondary data; however, we consider this tool a viable way of capturing the amount of information needed to assess a time series of nominal data on child vaccination in a short period of time. In addition, since the registry of AEFI in the EIR of the Juarez System is still recent, the availability of the data in the system can be improved, such as those referring to the clinical manifestations that needed to be searched in health reports. However, the system showed good sensitivity in the surveillance of AEFI and the results are consistent with the literature.

We conclude that there was no change in the safety profile of the basic scheme of vaccines in the period from 2000 to 2013. This study describes the use of EIR to better evaluate AEFI in a medium-sized municipality, highlighting its potential in the surveillance of these events. This information is relevant to the ongoing updating of vaccination standards in order to ensure the safety and reliability of the NIP, in addition to broadening the academic production on the subject. The intention is to contribute with the improvement of the EIR in Brazil, either for municipal initiatives of electronic registry with a vaccination component or for the implementation of the IS-NIP.

## Footnotes

a Ministério da Saúde (BR). Sistema de Informação do Programa Nacional de Imunizações –SI-PNI. Brasília (DF); s.d. [cited 2016 Jan 6]. Available from: http://pni.datasus.gov.br/

b Ministério da Saúde (BR), Secretaria de Vigilância em Saúde, Departamento de Vigilância das Doenças Transmissíveis. Manual de vigilância epidemiológica de eventos adversos pós-vacinação. 3. ed. Brasília (DF); 2014.
